# Gastroscopy in Pediatric Surgery: Indications, Complications, Outcomes, and Ethical Aspects

**DOI:** 10.1155/2015/820340

**Published:** 2015-03-25

**Authors:** Louise Roth, Martin Salö, Mette Hambraeus, Pernilla Stenström, Einar Arnbjörnsson

**Affiliations:** Department of Pediatrics, Clinical Sciences Lund, Lund University, 22185 Lund, Sweden

## Abstract

*Background.* The aim of this study was to map gastroscopies performed at a single tertiary pediatric surgery centre to investigate indications, complications, outcomes, and ethical aspects. *Material and Methods.* A retrospective study of gastroscopies performed during two time periods (2001–2004 and 2011–2014) was conducted. Data regarding indications, outcomes, and complications of pediatric gastroscopies were analysed from a prospectively collected database. *Results.* The indications for gastroscopies changed over time. Therefore, 376 gastroscopies performed from 2011 through 2014 were studied separately. The median patient was four years old. The predominant indications were laparoscopic gastrostomy (40%), investigation of gastroenterological conditions (22%), obstruction in the upper gastrointestinal tract (20%), gastroesophageal reflux disease (GERD) (15%), and other indications (3%). Percentages of gastroscopies with no positive findings for each condition were laparoscopic gastrostomy, 100%; gastroenterological conditions, 46%; obstruction in the upper gastrointestinal tract, 36%; GERD, 51%. Furthermore, gastroscopies did not lead to any further action or change in treatment in 45% of gastroenterological conditions and 72% of GERD cases. The overall complication rate was 1%. *Conclusion.* The results are valuable to educate pediatric surgeons and to inform health care planning when including gastroscopy within clinical practice.

## 1. Introduction

The use of gastroscopy has been growing worldwide, as well as its diagnostic and therapeutic possibilities [1]. Gastroscopy has been used in our Department of Pediatric Surgery since the early 1990s. Today, it is used on a daily to weekly basis to investigate gastroesophageal reflux disease (GERD), dysphagia with or without associated esophageal atresia, and gastroenterological disorders such as coeliac disease, during laparoscopic gastrostomies, and, more rarely, to detect foreign bodies [2–4]. Although endoscopy is used routinely by pediatric surgeons, no recent studies have evaluated gastroscopies within a pediatric surgery department.

Performing a gastroscopy on a child is associated with risks. The gastroscopy itself is associated with a risk of bleeding or perforation of the esophagus [5–8]. Moreover, in our hospital it is standard procedure that all children undergoing gastroscopy receive general anesthesia. The reason for this is that endoscopies in awake children may be poorly tolerated by the child and his or her guardians. Furthermore, several of our patients suffer from GERD, which increases the risk of vomiting and aspiration. Performing endoscopies in awake children may provoke these events. There are, however, several risks associated with general anesthesia, including dental injury, sore throat, postoperative nausea and vomiting (PONV), respiratory problems, and, in severe cases, cardiac problems including cardiac arrest [9–11]. Anesthetizing children may be associated with additional risks such as neurodevelopmental damage, but this has not yet been determined [12–14]. All these risks must be considered when deciding to perform a gastroscopy on a child.

The aim of this study was to map the routine use, outcomes, and ethical aspects of pediatric gastroscopy within the Department of Pediatric Surgery. The purpose was to be able to determine which gastroscopies have a favourable risk/benefit ratio. Our study sought to answer the following questions.

- Are any unnecessary gastroscopies performed? Should gastroscopy be used more or less frequently?
- What are the benefits for the children? Can outcomes be predicted?

The goal for our findings was to outline the volume of pediatric gastroscopies, their indications, changes in the frequencies of these indications over time, and their complications. This information is important for future decision-making in the clinical setting and for administrative planning.

## 2. Material and Methods

To identify the indications for the use of pediatric gastroscopy and patient outcomes, the prospectively collected database containing all children admitted to the Department of Surgery was used. This department is a tertiary centre that provides free public health care for an area of 1.8 million inhabitants and 22,000 newborns each year. Surgeons receive a monthly salary with overtime payment related to their time on call and time on duty doing surgical work.

### 2.1. Data Collection

Gastroscopies were identified in the database using surgery codes “gastroscopy” and “gastroscopy with biopsy” over two time periods: January 2001 to December 2004 and July 2011 to May 2014. Gastroscopies were sorted into five different types of indications:

1. obstruction in the upper gastrointestinal (GI) tract: dilatation and calibration of esophagus after surgery for esophageal atresia, after malignant disease, after esophagitis, and after intake of corrosives; dilatation and calibration of duodenum after duodenitis; investigation of obstruction in the pyloric region, in suspected high intestinal obstruction; investigation of suspected esophageal achalasia; and investigation of dysphagia;
2. gastroesophageal reflux disease (GERD): investigation of suspected or diagnosed GERD; preoperative investigation; endoscopy for postoperative complications; and examination to evaluate the outcome of fundoplication surgery;
3. investigation of gastroenterological conditions: investigation of suspected coeliac disease, inflammatory bowel disease, gastritis,* Helicobacter pylori* infection, bleeding from the GI tract, malabsorption, and intermittent abdominal pain;
4. laparoscopic gastrostomy: laparoscopic gastrostomy concurrent with an evaluating gastroscopy performed according to local routines [2];
5. other: treatment of oesophageal varices; removal of foreign bodies in the upper GI tract; and placement of capsule for endoscopic investigation.

Variables analysed included gender, age at examination, indication for the gastroscopy, whether or not a biopsy was taken, outcome, and complications related to the gastroscopy within 30 days.

### 2.2. Ethical Consideration

Intention to treat was the main analysis strategy and encompassed all patients. The study was performed according to the Declaration of Helsinki and was approved by the regional research ethics committee (registration number 2010/49). Data were anonymized prior to analysis and are presented in such a way that no single patient can be identified. Therefore, it was not necessary to obtain approval from patients' guardians. All evaluations, treatments, and procedures described in this paper were standard of care for patients and were conducted at the Department of Pediatric Surgery. No protocols that would have required appropriate informed consent or approval by an institutional review board were used. The ethical questions that may arise with gastroscopy in a child were considered according to the guidelines published in 2014 by the Swedish Council on Health Technology Assessment. The council published twelve questions in four different aspects of ethics to be used for consideration of whether a procedure is ethical [15].

### 2.3. Statistical Consideration

Because this was a descriptive study, no power calculations were performed. SPSS Statistics was used to compare data from the two time periods, 2001–2004 and 2011–2014, using a chi-squared test with an alpha level of 0.05.

## 3. Results

A total of 834 gastroscopies were performed from January 2001 through December 2004 and from July 2011 through May 2014. There was a statistically significant difference in the distribution of indications between the two time periods. The latter period was mapped in detail because it best represented current routines and should provide more accurate information on future routines. In total, 379 gastroscopies were performed in this period. Medical records were not accessible for three examinations, which were then excluded. Hence, the sample consisted of 376 examinations on 314 patients, of whom 289 had only one examination and 25 had multiple examinations.

### 3.1. Demographics

**Figure 1:**
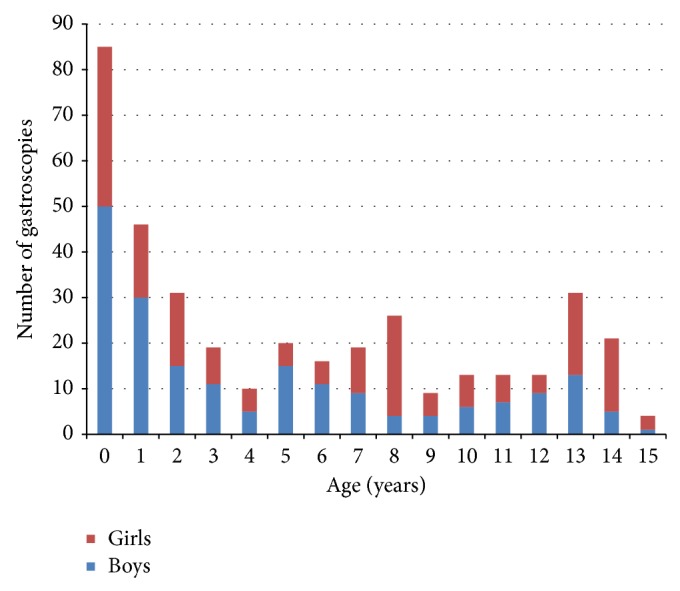
Age distribution across 376 gastroscopies performed from July 2011 to May 2014.

**Figure 2:**
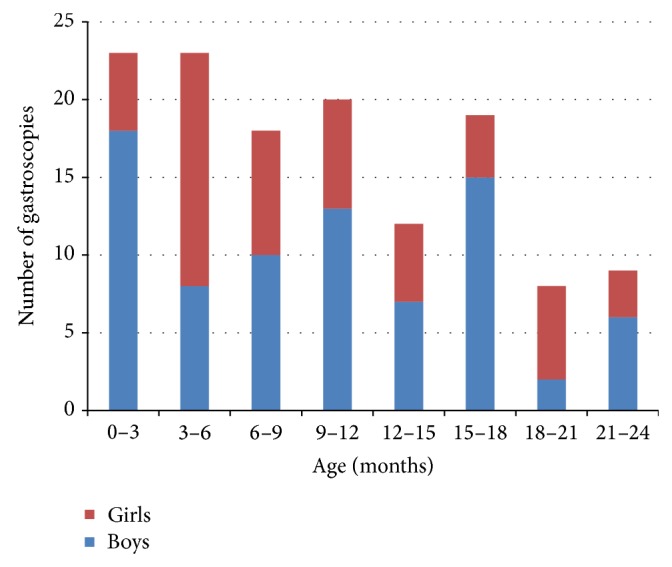
Age distribution across 132 gastroscopies performed on children under 2 years of age from July 2011 to May 2014.

The median age of patients was four years.  Figure 1 shows the age distribution for all gastroscopies and Figure 2 shows the age distribution for gastroscopies performed on children under two years of age. Of the 376 examinations, 195 (52%) were performed on boys and 181 (48%) on girls. Of the 314 patients, 168 (54%) were boys and 146 (46%) were girls.

### 3.2. Indications

**Figure 3:**
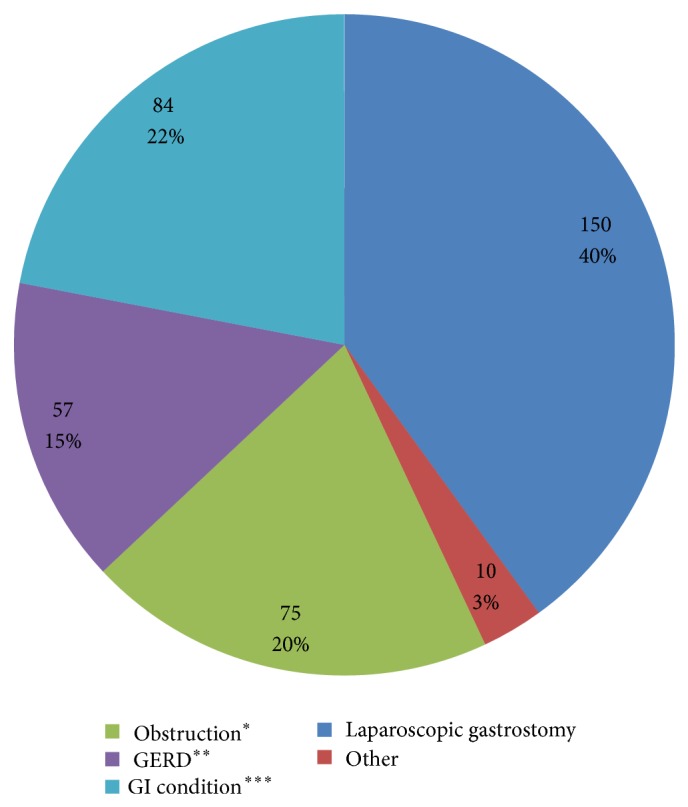
Distribution of indications for all gastroscopies performed on children from 0 to 15 years of age from July 2011 to May 2014. ^*^Obstruction in the upper gastrointestinal tract, ^**^gastroesophageal reflux disease, and ^***^investigation of gastroenterological conditions.

**Figure 4:**
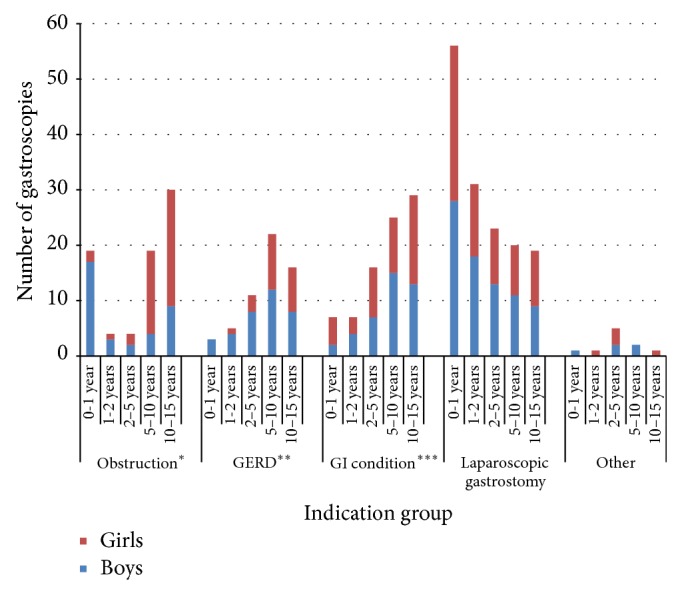
Age distribution among indication groups for all 376 patients from 0 to 15 years of age from July 2011 to May 2014. ^*^Obstruction in the upper gastrointestinal tract, ^**^gastroesophageal reflux disease, and ^***^investigation of gastroenterological conditions.

Figure 3 shows the distribution of indications of all gastroscopies performed during the study period. The most common indication was laparoscopic gastrostomy, followed by investigation of gastroenterological conditions, obstruction in the upper gastrointestinal tract, GERD, and other indications.  Figure 4 shows the gender and age distributions by indication.

### 3.3. Outcomes

Gastroscopies revealed no pathological findings in 51% of GERD cases, 46% of investigations of gastroenterological conditions, 36% of investigations of obstruction in the upper gastrointestinal tract, and 100% of laparoscopic gastrostomies. In the “other” group, all examinations were interventional and thus had pathological findings that were treated.

The proportion of examinations that did not lead to an action or change in treatment was 100% in the laparoscopic gastrostomy group, 72% in the GERD group, and 45% in the investigation of gastroenterological conditions group.  Table 1 shows the outcomes by indication, showing how patients benefitted from gastroscopy. The endoscopy performed after the insertion of a gastrostomy button using a laparoscopy-assisted technique disclosed the highest rate of normal findings. The gastrostomy button was found in place without intervening with passage through the stomach to the duodenum. Furthermore, any signs of GERD including esophagitis or hiatal hernia were not found. The children with severe GERD were already excluded from this group and had undergone an endoscopy for the work-up before antireflux surgery and for postoperative control and were included in another group. Further, some of the children receiving a gastrostomy suffered from metabolic diseases, malignancy, congenital heart disease, cystic fibrosis, and chronic kidney failure, hence, diseases not associated with GERD.

### 3.4. Complications

Of 376 examinations, complications related to the gastroscopy occurred four times (1%). Esophageal perforation occurred in two children, four and ten months of age. One child was examined with gastroscopy because of stricture after esophageal atresia and the other because of symptoms of GERD. Both were initially treated conservatively which was successful in one child, while the other child required a thoracotomy to drain the mediastinum. Aspiration and bronchial spasm occurred in two children, two and three years of age. Both were discharged from the hospital after 24 hours of uneventful observation in a pediatric surgical ward. Duration of hospital stay ranged from one day for the children with aspiration to 14 and 24 days for those with esophageal perforation. No lethal events occurred.

### 3.5. Comparison with Older Data

**Figure 5:**
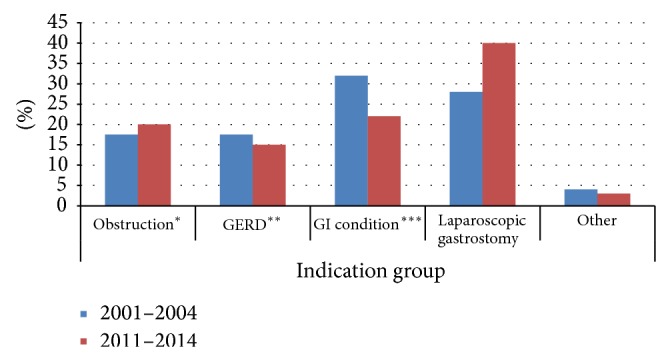
Percentage distribution of indications from January 2001 to December 2004 (455 patients) compared to July 2011 to May 2014 (376 patients). ^*^Obstruction in the upper gastrointestinal tract, ^**^gastroesophageal reflux disease, and ^***^investigation of gastroenterological conditions.

The use of gastroscopy increased from an average of 114 per year from 2001 to 2004 to an average of 129 per year from 2011 to 2014 (*P* = 0.0001). The distribution of indications also changed significantly between these time periods, as shown in Figure 5 (chi-square test: *P* < 0.001). For example, the use of gastroscopy to investigate gastroenterological conditions decreased from 31.9% to 22.3%, and gastroscopy used during laparoscopic gastrostomy increased from 27.7% to 39.9%.

### 3.6. Ethical Aspects

The four aspects of ethics outlined by the Swedish Council on Health Technology Assessment in 2014 [15] are considered in detail by the application of the ethical guidelines on gastroscopy in children posing the four questions and answers.

1. *What are the risks/benefits of the procedure?* The risk includes perforation or bleeding when dilating or picking up foreign objects and treating varices and risks associated with general anesthesia. The benefits include dilatation of esophageal strictures leading to improvement of dysphagia, inspection that excludes disease, chance to discover and exclude esophagitis at an early stage and start treatment, thereby reducing the risk for stricture and malignant disease, helpful when deciding if fundoplication is appropriate, chance to discover treatable disease and exclude severe disease [16], opportunity to establish correct tube placement, chance to discover esophagitis, gastritis, and insufficient cardiac function [2], removal of foreign objects, treatment of esophageal varices, enabling endoscopic capsule investigation in patients that cannot swallow the capsule, and calming guardians [4, 6, 7].
2. *Is use compatible with ethical values?* Every child is supplied with gastroscopy when there is a proper indication. It is fairly easy to objectively decide which patients fit into this indication group. In some cases, it is difficult to objectively determine whether a gastroscopy is necessary. Pressure from the patient's guardians might have an impact on the decision. It is unlikely with a positive finding if symptoms are diffuse [17].
3. *Are there reasons to believe that equal supply to this or other methods will be hindered if the method is in use?* This is unlikely, as there are no limitations as to how many children can undergo gastroscopy. Although, the practitioner's judgment could lead to unequal supply if physicians differ in how much they are affected by guardians' opinions. Also, depending on those performing the endoscopy the results are valued differently [18].
4. *Can the use of the method affect long-term consequences?* Yes, if a treatable disease is not discovered, it might not be treated which can aggravate the condition. If an intervention is necessary and succeeds it might mean that serious illness is avoided [12–14, 19].

## 4. Discussion

This study demonstrates that there has been an increase in the number of gastroscopies performed as well as a change in the distribution of indications for gastroscopies over the past 10–15 years. Furthermore, laparoscopic gastrostomy was the predominant indication for gastroscopy. The outcome of gastroscopy was negative in many cases; that is, no pathological findings were reported. Additionally, in the GERD group, the vast majority of examinations did not lead to any further action or change in treatment. This trend was also observed to a lesser extent in the investigation of gastroenterological conditions group. The overall complication rate was 1%, and no lethal events occurred.

### 4.1. Bias

The current study is a unique mapping of gastroscopies performed in pediatric surgery care for several reasons. First, the country's health care system is entirely free of cost for all children, which means that the sample represents all socioeconomic groups. Furthermore, the surgeons at the hospital are on salary and are not reimbursed based on the number of procedures they perform, which differs from some other countries, which often use the “fee for service” method [16]. This means that this study was unlikely to be affected by dropouts due to socioeconomic effects or bias due to economic interest. In this study, three examinations out of 379 were excluded, and the reason for exclusion in all three cases was because the medical records were inaccessible. Because of the modest amount of dropouts this was not considered a potential bias. However, this study has a distribution of case load with a strong bias to surgical problems. The gastroscopies were performed at a Department of Pediatric surgery where follow-up of previously operated gastrointestinal malformations is conducted which may influence the result. Since the surgeons want good results some pathology may be foreseen, which could be a bias.

### 4.2. Demographics

The median age in the study group was four years. Recent studies on pediatric gastroscopy have reported a mean age of 6.9–9 years [5, 17, 20, 21]. The use of mean age suggests normal distribution of patients' ages, which was not seen in the current study. In our study, 22% of patients (*n* = 85) were under one year of age, compared to another study that reported only 6.6% of patients being younger than one year of age [18]. The reason for the low median age in the current study is to be found in the numerous gastroscopies performed on children under two years of age for an indication of laparoscopic gastrostomy. The high number of laparoscopic gastrostomies at such an early age (median age one year) differs from a recent large cohort study where the median age was 2.66 years [22]. In contrast, a study on laparoscopic gastrostomies had a mean age of 21 months for placing of gastrostomy tubes and suggested that gastrostomy tube feeding might be introduced too late in some children [23]. A possible explanation for this diversity might be the different routines for laparoscopic gastrostomy across centres, which is not explored further in this study.

The group with an indication of obstruction in the upper GI tract had its first peak at age 0-1 year. This peak within the first year of life is not very surprising, since stricture as a complication of surgery for esophageal atresia is not unusual within the first months after surgery [6, 24]. However, the current study suggests that males within the first year of age suffer from more frequent complications, such as strictures. Certainly, the incidence of esophageal atresia is slightly higher among males but other studies do not suggest increased complications in males [19, 24]. Esophageal atresia is a rare condition, which means that only a small number of patients were treated for this condition in the current three-year study period.

The current study also showed peaks in girls at age 5–10 years and 10–15 years. There is no explanation to these peaks to be found in the literature. However, our study included two female patients in these age groups who each underwent numerous gastroscopies, one of whom suffered from a corrosive esophageal stricture and one had an esophageal stricture due to malignant disease. This implies that the gender differences in both cases above could be explained by skewing of results by individual patients due to a relatively small number of examinations [6, 7, 24].

### 4.3. Distribution of Indications

To our knowledge, no other investigations have been published recently on the indications for gastroscopy in pediatric surgery departments. Thus, there is no basis to compare the current study with previous results. At our centre, laparoscopic gastrostomy was the predominant indication, followed by investigation of gastroenterological conditions, obstruction in the upper gastrointestinal tract, GERD, and other indications.

Studies that have mapped gastroscopies at pediatric hospitals have shown diverse results. A retrospective study from 2014, which analysed all gastroscopies performed at a pediatric tertiary centre in Pakistan over two years, identified the most common indication as failure to thrive with suspected coeliac disease (31%) [20]. Another retrospective study from 2004, which examined all gastroscopies performed on children at a university hospital in Saudi Arabia over ten years, found the most common indications to be duodenal biopsy (29%) and abdominal pain (24%) [25]. In the current study, all three of these indications would have been grouped under investigation of gastroenterological conditions, which was not close to being the most common indication in the current study. However, these previous investigations were conducted among children in nonwestern countries. It is possible that these cohorts of children have different disease panoramas and that the organization and economic situations are different from and, thus not comparable to, those in the Swedish population.

A study from 2010 examined the changing indications of gastroscopy over a 20-year period in the US. Children that had undergone their first gastroscopy with biopsy were included. Abdominal pain was the predominant indication at 43% [21]. In the current study, this indication would have been included within gastroenterological conditions, which only accounted for 22% of gastroscopies. A similar study from 2013 at a children's hospital in the US examined the diagnostic yield of first-time pediatric gastroscopies with biopsies. The most common indications were generalized abdominal pain (29%), GERD (12%), and failure to thrive (10%) [18]. The current study had a similar proportion of GERD (15%) but less frequent abdominal pain and failure to thrive. However, these two studies excluded all repeated examinations within patients and gastroscopies performed without biopsies, which makes it complicated to compare to the current study.

To sum up, the results of the current study concerning the distribution of indications are quite different from previous studies. A likely explanation is that the studies presented above included mostly gastroenterological, and not surgical, patients. Overall, the number of gastroscopies performed within a gastroenterology department is much higher than the number performed within a surgery department. Thus, when mapping gastroscopies at a pediatric hospital, data from pediatric surgery patients are likely to be obscured by the much greater volume of data from gastroenterological patients. Consequently, the results of the pediatric surgery patients alone cannot be seen. It is noteworthy that no study in the 21st century has mapped gastroscopies within a pediatric surgery department.

### 4.4. Outcome

Of the gastroscopies performed for an indication of GERD, the majority had no positive findings and almost three quarters did not lead to any further action or change in treatment. For investigation of gastroenterological conditions, almost half of the examinations had no positive findings and almost as many did not lead to any further action or change in treatment. For laparoscopic gastrostomy, there was not a single pathological finding. This amount of negative gastroscopies is remarkable, and even though several other studies present similar results [16–18, 20, 21, 25], this compels practitioners to consider a more conservative approach than gastroscopy for these indications.

Before deciding to perform a gastroscopy examination, the physician should consider the potential outcomes and how the patient might gain from the examination. This information will help the practitioner to analyse the risk/benefit ratio. In this consideration, it is crucial to consider risks associated with performing a gastroscopy and the ethical aspects of the procedure. There is a risk not only of medical complications, but also of negative mental impact on the child. Considering possible gain, if the aim is to confirm a suspected condition, the practitioner must ask whether the examination will change the patient management. In many cases, the patient has started treatment for the condition before the gastroscopy is conducted. And if the diagnosis is verified, there will be no change in treatment. This is the case for many patients with GERD. In this group, it might be possible to cut down on gastroscopies in order to not subject children to the risks of a gastroscopy unless it is necessary.

Naturally, in some of the cases mentioned above, the gastroscopy is performed to rule out severe conditions such as malignancies. These examinations are of great value even though there is no positive outcome and they do not lead to further action. However, these conditions are very rare in pediatric patients and the explanation would not account for all of the above examinations. In fact, some “unnecessary” gastroscopies are performed in response to pressure from the patients' guardians or referring doctor, not necessarily because the pediatric surgeon considered the examination obligatory [17]. On the other hand, it should be noted that a negative outcome or no action does not always imply that the examination was unnecessary. An endoscopic mapping with negative results can calm the patient, guardians, and/or referral doctor and prevent further examination. However, the question always remains: is it worth the risk?

To our knowledge, this is the first study published to apply the ethical guidelines as outlined by the Swedish Council on Human Technology Assessment. Thus, it cannot be compared with other studies in this respect. Application of these guidelines did not identify any ethical issues necessitating an immediate change in the use of gastroscopy in pediatric surgical practice.

### 4.5. Complications

In this study, complications occurred in four of 376 (1%) gastroscopies. Two of the complications were esophageal perforations and could be considered as major. One of these had to undergo surgical treatment and one had a hospitalization prolonged by several weeks. We could find no prior studies that mapped gastroscopy complications at a pediatric surgery department. A study of over 10.000 pediatric gastroscopies from 2007, where pediatric gastroenterologists reported the results, identified a complication rate of 2.3%. The authors reported the most common “immediate complications” to be hypoxia (1.5%) and bleeding (0.3%). No deaths or perforations were reported [5]. Unfortunately, this study did not report the hypoxia duration or sequel and did not define “immediate complications.” For example, the most severe complication in the current study was not discovered until two days after the examination and might not have been included within “immediate complications” and, therefore, would not have been reported. Furthermore, gastroscopies performed at a gastroenterology unit might not be comparable since they are less interventional and cover a different spectrum of measures. For example, studies on treatment of esophageal strictures in smaller study populations often report perforation as a complication event [6, 7].

### 4.6. Development over Time

Over time, there was a statistically significant increase of the annual number of gastroscopies, from an average of 114 per year in 2004 to 129 per year in 2014. The increase is supported by another study that reported a 12-fold increase in the number of gastroscopies performed over a ten-year period [21]. There was also a statistically significant difference in the distribution of indications for gastroscopies between the two time periods (2001–2004 and 2011–2014) studied. The largest changes were a decrease in investigation of gastroenterological conditions and an increase in laparoscopic gastrostomy. There is a plausible explanation for why more gastroscopies were performed to investigate gastroenterological conditions ten years ago. The European Society for Paediatric Gastroenterology, Hepatology, and Nutrition published new guidelines for the diagnosis of coeliac disease in 2012. The new guidelines state that gastroscopy is not always necessary for diagnosis, unlike the previous guidelines [26]. Moreover, even before the new guidelines were published, clinical practice had started to change at our centre, and biopsy was not necessary for diagnosis. This resulted in fewer gastroscopies being performed on patients with significant clinical symptoms and elevated serum markers, a development that started before 2011 [27]. The considerably greater amount of gastroscopies performed with the indication laparoscopic gastrostomy in 2011 through 2014 than in 2001 through 2004 is noted. The current study did not investigate the reason for this increase further.

## 5. Conclusion

There has been an increase in the number of gastroscopies performed the past years and the indications have changed. There was a modest diagnostic yield of gastroscopy to investigate GERD or gastroenterological conditions. Furthermore, gastroscopies performed in association with laparoscopic gastrostomy surgery did not have any positive findings. The overall complication rate was 1%. The implications of this study are that practitioners and administrators in each patient's case should carefully consider whether a gastroscopy examination is necessary. The ethical analysis did not necessitate a change in the use of this procedure but should be considered in deliberations on pediatric gastroscopy across different indications.

## Figures and Tables

**Table 1:** Summary of the numbers and types of outcomes in the five indication groups.

Indication group	Outcome, percentage of patients (*n*)
Obstruction in upper gastrointestinal tract	(i) Dilatation of stenosis, 55% (41) (ii) Calibration, 29% (22) (iii) Inspection with positive findings, 8% (6) (iv) Inspection with negative findings, 7% (5) (v) Mapping before atresia surgery, 1% (1)

Gastroesophageal reflux disease	(i) No positive findings at either of the following aspects: inspection of mucosa/inspection of cardiac function/biopsy/24-hour pH measurement, 51% (29), of which 10% (3) led to dose change in proton pump inhibitor (PPI) treatment and 90% (26) led to no change of treatment (ii) Positive findings at one or more of the following aspects: inspection of mucosa/inspection of cardiac function/biopsy/24-hour pH measurement, 47% (27), of which 30% (8) led to starting up with/dose escalation of PPI, 7% (2) led to decision of fundoplication, 7% (2) led to further investigation, and 56% (15) led to no action taking/no change of treatment (iii) Calibration, 2% (1)

Investigation of gastroenterological conditions	(i) No positive findings at inspection/biopsy, 46% (39), of which 23% (9) led to further investigation/treatment with gluten-free diet and 77% (30) led to no action taking/no change of treatment (ii) One or more positive findings at inspection/biopsy, 50% (42), of which 69% (29) led to treatment with gluten-free diet/PPI/eradication treatment of helicobacter pylori/total parenteral nutrition, 12% (5) led to further investigation, and 19% (8) led to no action/no change of treatment (iii) Inconclusive findings, 4% (3)

Laparoscopic gastrostomy	(i) Inspection of the placement of gastrostomy and signs of esophagitis, 100% (150) (of which none showed signs of esophagitis)

Other	(i) Removal of foreign body, 60% (6) (ii) Rubber band ligation of varices, 20% (2) (iii) Placement of capsule for video endoscopic examination, 20% (2)
